# Retrospective analysis of incidental pulmonary nodules detected on routine chest CT scans

**DOI:** 10.6026/973206300213067

**Published:** 2025-09-30

**Authors:** Mimithira Thiyagarajan, Sudeeshna Thiyagarajan, Shaheed Shaik, Satya Naga Shravan Chilukuri, Prashant Sundeep Gupta, Nadhu Krishna Maheswaran Nair

**Affiliations:** 1 Department of Emergency Medicine, PSP Medical College Hospital and Research, Tamil Nadu, India; 2 Department of Critical Care and ER, CMC Vellore, Tamil Nadu, India; 3 Department of General Medicine, Gandhi Medical College, Secunderabad, Telangana, India; 4 Department of Internal Medicine, Junior Clinical Fellow in Medicine, Swansea Bay University, Swansea, Wales United Kingdom; 5 Department of Medicine, Surat Municipal Institute of Medical Education and Research, Gujarat, India; 6 Department of Internal Medicine, Radiology, Emergency Medicine, GG Hospital, Kerala, India

**Keywords:** Incidental pulmonary nodules, chest CT, retrospective analysis, lung cancer risk, nodule follow-up, radiologic features

## Abstract

The characteristics and outcomes of incidental pulmonary nodules (IPNs) identified on routine chest CT scans in 138 adult patients over a 24-month period is of interest. Nodule features including size, location, morphology and growth were analyzed alongside clinical follow-up data. The majority of nodules were benign and stable, with only 7.2% showing suspicious features warranting further investigation. Smoking history and spiculated margins were significantly associated with malignant risk. Thus, we show the importance of standardized follow-up and risk stratification protocols for incidental pulmonary nodules.

## Background:

The increasing use of computed tomography (CT) for screening has led to a rising number of incidental pulmonary nodule detections. The incidental pulmonary nodule detection rates are well-documented in Western populations, but large-scale data from Chinese cohorts remain limited [1]. Incidental pulmonary nodules (IPNs) are frequently identified on routine chest computed tomography (CT) scans conducted for various non-pulmonary indications, owing to the widespread use of imaging in clinical practice [2]. With reported detection rates ranging from 20% to 50%, the clinical relevance of these nodules often presents a diagnostic challenge, particularly in distinguishing benign findings from early-stage malignancies [3]. Most nodules are ultimately benign, resulting from prior infections, granulomatous disease, or environmental exposures [4]. However, a small but critical subset may represent early lung cancers and timely identification of these can significantly improve patient outcomes. Radiological characteristics such as nodule size, location, margin definition, growth pattern and density are important in risk stratification and follow-up planning [5]. Patient-related factors-especially age, smoking history and comorbidities-also contribute to malignancy risk estimation [6]. Despite available guidelines like those from the Fleischner Society, real-world adherence to nodule management recommendation remains variable [7]. This retrospective analysis aims to evaluate the prevalence, radiologic features and clinical outcomes of incidental pulmonary nodules detected on routine chest CT scans [8]. Therefore, it is of interest to enhance understanding of IPN patterns, guide appropriate follow-up, and identify features that warrant closer surveillance or further diagnostic work-up.

## Materials and Methods:

This retrospective study was conducted at a tertiary care hospital by analyzing chest computed tomography (CT) scans performed between \ January 2022 and December 2023. Adult patients aged 18 years and above who underwent routine chest CT for non-pulmonary and non-oncologic indications (such as trauma evaluation, preoperative assessments, or infection screening) were screened. A total of 138 patients were included based on the incidental detection of at least one pulmonary nodule not previously known or under investigation. Patients with a history of primary lung malignancy, metastatic disease, or documented prior pulmonary nodules were excluded from the study. Radiological data were extracted from the hospital's imaging archives and included detailed assessment of each nodule's characteristics-size (measured in the largest axial diameter), number, location (upper vs. lower lobes), density (solid, ground-glass, or subsolid), margins (smooth, lobulated, or spiculated), and presence of calcification or cavitation. Patient demographics, clinical history including smoking status, and presence of comorbidities such as COPD, tuberculosis, or immunosuppression were obtained from electronic medical records. Follow-up imaging reports and histopathological outcomes (if available) were reviewed to assess changes in nodule size or features over time. Nodules were stratified by size: <6 mm (small), 6-8 mm (intermediate), and >8 mm (large). Morphological features and growth on subsequent scans were used to assess risk for malignancy. The primary outcome of interest was the proportion of nodules requiring further diagnostic evaluation, such as biopsy or PET-CT. Statistical analyses included descriptive summaries and evaluation of associations between clinical or imaging features and suspicious/malignant outcomes using chi-square and logistic regression, with a significance threshold of p<0.05.

## Results:

Among 138 patients with incidental pulmonary nodules (IPNs), the majority of nodules were small, benign, and remained stable on follow-up imaging. However, specific radiologic features-such as spiculated margins, upper lobe location, and larger size-were significantly associated with a higher likelihood of further evaluation or malignancy. Smoking history also showed a strong correlation with nodules deemed suspicious. Table 1 (see PDF) shows most patients were middle-aged males, with a significant proportion having a history of smoking and comorbidities such as COPD and prior tuberculosis. Table 2 (see PDF) shows the majority of nodules were small and solid, with a minority showing suspicious morphology such as spiculation or lobulation. Table 3 (see PDF) shows Nodules larger than 8 mm were more likely to undergo follow-up imaging and diagnostic workup. Table 4 (see PDF) shows spiculated margins and upper lobe location were significantly associated with biopsy or advanced imaging. Table 5 (see PDF) shows patients with smoking history were more likely to have nodules recommended for biopsy or close imaging follow-up. Table 6 (see PDF) shows among nodules followed over time, most remained stable; a few showed growth or malignant progression. Table 7 (see PDF) shows all confirmed malignancies were >8 mm, spiculated, and located in upper lobes; all occurred in smokers. Table 8 (see PDF) shows nodules with ground-glass or subsolid appearance showed more frequent recommendation for monitoring. Table 9 (see PDF) shows most clinicians adhered to Fleischner Society guidelines in management, especially for nodules ≥6 mm. Table 10 (see PDF) shows the overall prevalence of malignancy among incidental nodules in this study was low (2.2%).

## Discussion:

This retrospective study provides valuable insights into the prevalence, radiologic features, and clinical management of incidental pulmonary nodules (IPNs) detected on routine chest CT scans. Most nodules were small (<6 mm), solitary and had benign imaging characteristics such as smooth margins and solid density. These findings are consistent with previous literature indicating that the majority of IPNs are non-malignant and often require no intervention beyond observation [8]. However, certain radiologic features-including size >8 mm, spiculated margins, subsolid or ground-glass morphology, and upper lobe location were significantly associated with increased likelihood of further diagnostic evaluation, including PET-CT or biopsy [10]. This aligns with current guidelines and supports the utility of morphological assessment in risk stratification. Particularly, all nodules found to be malignant in our cohort exhibited a combination of these high-risk characteristics and occurred exclusively in current or former smokers, highlighting the additive value of clinical context in nodule evaluation [9]. Importantly, our findings also emphasize the role of smoking history as a critical determinant of management. Patients with a history of tobacco use were more likely to undergo further investigations and had a higher prevalence of suspicious nodule features. This underscores the importance of incorporating smoking status into risk models to optimize follow-up protocols and reduce unnecessary procedures in low-risk individuals [10]. The growth behavior of nodules over time provided reassurance regarding the low malignancy rate-only 4.9% of nodules with follow-up imaging showed progression, and 2.2% were ultimately diagnosed as malignant. These outcomes reinforce the safety of guideline-based surveillance strategies and the low overall risk associated with incidentally detected nodules, especially when size and imaging features are favorable [11]. Clinical adherence to Fleischner Society guidelines was high in this study, indicating growing standardization in radiology-driven recommendations. Nonetheless, variability still existed, particularly in nodules smaller than 6 mm or those with ambiguous morphology. Ensuring uniform application of evidence-based protocols remains essential to prevent over-investigation and patient anxiety [12]. Overall, this study confirms that while incidental pulmonary nodules are common, only a small subset carry malignant potential. A structured approach based on nodule size, morphology, and patient risk factors-particularly smoking history can facilitate appropriate follow-up while avoiding unnecessary interventions. Further prospective studies and integration of AI-based risk calculators may refine predictive accuracy and support individualized decision-making in pulmonary nodule management.

## Conclusion:

Incidental pulmonary nodules are frequently detected on routine chest CT scans, with the majority being benign and requiring minimal intervention. However, nodules larger than 8 mm, those with spiculated margins, upper lobe location, or subsolid morphology-especially in patients with a history of smoking-warrant closer evaluation due to increased malignancy risk. Adherence to guideline-based follow-up protocols is crucial for balancing early cancer detection with the avoidance of unnecessary procedures. A structured, risk-stratified approach enhances clinical decision-making and improves the efficiency of incidental nodule management.
